# Insufficient autophagy relates to mitochondrial dysfunction, organ failure and adverse outcome in an animal model of critical illness

**DOI:** 10.1186/cc10618

**Published:** 2012-03-20

**Authors:** J Gunst, I Derese, A Aertgeerts, EJ Ververs, A Wauters, G Van den Berghe, I Vanhorebeek

**Affiliations:** 1 Katholieke Universiteit Leuven, Belgium

## Introduction

Increasing evidence implicates mitochondrial dysfunction in the pathogenesis of critical illness-induced multiple organ failure. We previously demonstrated that prevention of hyperglycemia limits mitochondrial damage in vital organs [1,2], thereby reducing morbidity and mortality [3]. We now hypothesize that inadequate activation of mitochondrial repair processes (mitochondrial clearance by autophagy, mitochondrial fusion and fission, and biogenesis) may contribute to accumulation of mitochondrial damage, persistence of organ failure and adverse outcome of critical illness.

## Methods

We addressed this hypothesis in a rabbit model of critical illness. First, we studied whether vital organ mitochondrial repair pathways are differentially affected in surviving and nonsurviving hyperglycemic animals, in relation to mitochondrial and organ function. Next, we investigated whether preventing hyperglycemia with insulin affects mitochondrial repair over time. We quantified mRNA/protein levels of key players of these processes. Activities of respiratory chain complexes I to V were measured spectrophotometrically. Plasma transaminases and creatinine were measured as markers of liver, respectively kidney, dysfunction.

## Results

In the liver and kidney of nonsurviving hyperglycemic rabbits, molecular markers of insufficient autophagy were evident, including accumulation of p62 protein (but no increase of p62 mRNA) and decreases in the autophagosome-associated protein LC3-II (microtubule-associated protein light chain 3). These changes were less prominent in surviving animals and correlated with impaired mitochondrial and organ function. In contrast, key players in mitochondrial fusion, fission or biogenesis were not affected by survival status. Therefore, we focused on autophagy to study the impact of preventing hyperglycemia. Both after 3 and 7 days of illness, autophagy was better preserved in normoglycemic than in hyperglycemic rabbits, which correlated strongly with improved mitochondrial and organ function.

## Conclusion

These findings put forward insufficient autophagy as a potentially important contributor to mitochondrial and organ dysfunction in critical illness, and open perspectives for therapies that activate autophagy during critical illness.
